# Juvenile idiopathic arthritis-associated uveitis in the era of biological therapy: how the disease changed in more than 20 years of observation in a tertiary referral center in Rome (Italy)

**DOI:** 10.1007/s10792-021-02043-1

**Published:** 2021-10-20

**Authors:** E. Del Giudice, C. Simio, A. Scala, A. Di Coste, G. La Torre, L. Spadea, R. Lubrano, M. Duse, M. P. Paroli

**Affiliations:** 1 Department of Maternal Infantile and Urological Sciences, Sapienza University of Rome, Polo Pontino, Italy; 2 Department of Sense Organs, Eye Clinic, Uveitis Unit, Sapienza University of Rome, V.le del Policlinico 155, 00161 Rome, Italy; 3 Department of Maternal Infantile and Urological Sciences, Sapienza University of Rome, Rome, Italy; 4 Department of Public Health and Infectious Diseases, Sapienza University of Rome, Rome, Italy

**Keywords:** Juvenile idiopathic arthritis, Uveitis, Ocular complications, Visual prognosis, Biological therapy

## Abstract

**Objectives:**

To describe the ophthalmological characteristics in a Juvenile idiopathic arthritis (JIA) cohort and to evaluate how therapeutic advances have changed the course of the uveitis.

**Methods:**

Analysis of a retrospective cohort study of consecutive JIA pediatric patients including JIA-associated uveitis (JIA-U) and comparison with a previous study in the same uveitis center assessed before the wide-spread of biological therapy.

**Results:**

The total of 49 JIA patients were analyzed, of whom 18 JIA-U, compared with a JIA-U past cohort of 66 patients. Systemic corticosteroids were used significantly less in the current JIA-U group (*p* = 0.008) than in the past one. JIA-U present cohort was on therapy more frequently with conventional synthetic disease-modifying anti-rheumatic drugs (csDMARDs) than the past group (*p* = 0.039), mostly treated with methotrexate (93.3%). Furthermore, a larger use of biologic disease-modifying anti-rheumatic drugs (bDMARDs) was described in the current JIA-U group (*p* = 0.005) also associated with csDMARDs (*p* = 0.003). Adalimumab was used more (72.7%) in the present JIA-U cohort compared to a larger treatment with infliximab (61.5%) in the past (*p* = 0.005). Higher number of uveitis recurrences was observed in the previous cohort compared to the current one (*p* = 0.005). Fewer complications were described in this study than in the previous: posterior synechiae (*p* = 0.007), cataract (*p* < 0.001), band keratopathy (*p* < 0.001), and elevated intraocular pressure (IOP) (*p* = 0.047).

**Conclusion:**

Current therapies reduced the uveitis recurrences and ocular complications including cataract due also to the lower use of corticosteroids. The new close collaboration with the pediatric rheumatologic center in the same University has contributed to the care improvement and decrease of uveitis complications.

## Introduction

JIA is the most common chronic rheumatic disease in children with an incidence of 8.2 (7.5–9.0)/100000 cases under 16 years of age, and an annual prevalence of approximately 70.2 (16–140)/100000 [1]. Chronic anterior uveitis (CAU) is the most frequent extra-articular manifestation of JIA and its incidence is thought to be approximately 1/10000 with some evidence that it is less frequent in Asian populations affected by JIA [2, 3].

The reduction of visual acuity is mainly due to complications resulting from CAU as well as side effects owed to local steroid drugs used to contrast it [4]. The main risk factor for visual impairment is chronic intraocular inflammation, therefore all the therapeutic options are aimed to reduce or to resolve it. Nevertheless, the management strategies of JIA-related uveitis remain a relevant clinical challenge [5].

The first-line standard therapy is topical corticosteroid, even though up to 60% of the children need further therapies to achieve quiescence [6]. Sometimes systemic corticosteroids are also used for a short time in severe disease, but the next step is usually the immunosuppressive therapy, as disease-modifying anti-rheumatic drugs (DMARDs), mainly using methotrexate (MTX), or other immunosuppressive drugs in MTX-resistant cases [6]. This therapy is usually able to solve the recalcitrant cases achieving a good control of the uveitis in approximately 70–80% of patients within 3–4 months [7, 8]. However, if the DMARDs are not effective, the biological therapies are considered an appropriate third-line treatment [4]. Currently, TNF-α blocking agents are the main biologics used as treatment of chronic childhood uveitis although in unresponsive JIA uveitis cases other biological drugs options are available [9–12].

The aim of the study was to describe the characteristics of a JIA pediatric population and to assess how therapeutic advances have changed the uveitis course by comparing with a previous cohort evaluated before the wide-spread use of biological therapy in the same uveitis center.

## Methods

### Patients and study design

We conducted a retrospective analysis of all consecutive pediatric patients with suspected diagnosis of JIA referred to the Department of Pediatrics and to the Department of Sense Organs, Eye Clinic, Uveitis Unit (Policlinico Umberto I, Sapienza University of Rome), from April 2012 to July 2018. Patients with diagnosis of JIA according to the International League of Associations for Rheumatology criteria were eligible to participate [12, 13]. The whole cohort was subdivided into two groups: patients with only JIA (group I) and patients with JIA-associated uveitis (JIA-U) (group II). Moreover, data about the JIA-U group were compared to a previous JIA-U cohort (group III) observed from July 1995 to March 2012 in the same referral uveitis center already reported in a published study [14].

Following the close collaboration with the pediatric rheumatologic center in the same University, all JIA patients were assessed by a pediatric rheumatologist and an ophthalmologist at JIA diagnosis and then at every follow-up. All examinations were performed in the same center and by the same group of physicians.

Active uveitis was defined when at least one eye showed an anterior chamber (AC) cells grade above 0 according to the SUN (Standardization of Uveitis Nomenclature) criteria as indicated by the ophthalmologist [15].

Each patient was observed according to the ophthalmological screening uveitis risk for JIA, whereby checks depend on age at disease onset, ILAR reference category, ANA (antinuclear antibodies) positivity, and disease duration [10, 16, 17].

Demographic data and disease-related variables including sex, JIA disease subtype according to ILAR category [12], age at JIA diagnosis and uveitis diagnosis, JIA and uveitis disease duration, were assessed for each patient.

The JIA uveitis’s characteristics and visual acuity were described: date of uveitis diagnosis, anatomical involvement (anterior, intermediate, posterior, panuveitis), laterality, as well as presence of ocular complications and rates of uveitis relapses. Moreover the activity ocular indices were collected for each affected eye according to the SUN International Working Group: the total number of AC (anterior chamber) cells, AC cell grade before and after pupil dilatation (grade 0 (< 1 cells), grade 0.5 + (1–5 cells), 1 + (6–15 cells), 2 + (16–25 cells), 3 + (26–50 cells), 4 + (> 50 cells) [15, 18]. Each patient underwent a routine laboratory examination at the first examination and during follow-up. According to clinical history, JIA subtypes and uveitis features, other serological, and immunological exams were analyzed, such as erythrocyte sedimentation rate [ESR], C-reactive Protein [CRP], and ANA [antinuclear antibodies]).

Furthermore, data about the use of conventional synthetic disease-modifying anti-rheumatic drugs (csDMARDs), biologic disease-modifying anti-rheumatic drugs (bDMARDs), or a combination therapy (csDMARD plus bDMARD) were analyzed. Information about the csDMARD used (methotrexate [MTX], azathioprine [AZA], salazopyrine [SSZ]) and bDMARD (adalimumab, infliximab, etanercept, tocilizumab, abatacept), as well as concomitant treatment with mydriatic drops and corticosteroids (topical and/or systemic), non-steroidal anti-inflammatory drugs (NSAIDs), mean therapy duration and mean time between the introduction of csDMARD and the bDMARD were also recorded.

According to the current guidelines, the first-line treatment approach was the use of topical steroids and mydriatics, then systemic immunosuppressive therapy (mostly methotrexate), and, in case of limited clinical response, the additional use of a biological agent, largely TNF alfa blockers. In case of inadequate response to one biological agent, different possibilities of switch were considered according to the literature [4, 10].

All JIA patients underwent ophthalmological examinations following the international recommendations: high-risk children screening every three months (oligoarthritis, polyarthritis rheumatoid factor negative, psoriatic arthritis, or undifferentiated arthritis who are also antinuclear antibody (ANA) positive, younger than 7 years of age at JIA onset, and have JIA duration of 4 years or less); low or moderate risk children screening every 6–12 months (those with high-risk JIA categories but with ANA negative, age of 7 years or older at JIA onset, or have JIA duration of more than 4 years, or systemic JIA or polyarthritis rheumatoid factor positive or enthesitis-related arthritis) [10].

JIA-U patients were then examined at variable interval time depending on therapeutic response and uveitis severity.

The ophthalmological examination included measurement of BCVA (best correct visual acuity) with Snellen’s chart at 3 m; examination of the anterior segment with slit-lamp biomicroscopy and intraocular pressure assessment using Goldmann applanation tonometer; fundus examination using bilateral indirect ophthalmoscope. The evaluation of ocular inflammation was based on the cellular activity in the anterior chamber (PK, cells, and flare) according to SUN criteria. Some patients underwent further investigations such as spectral-domain optic coherence tomography (OCT)*,* retinal fluorangiography (FA), and indocyanine angiography (ICGA) when required by the course of uveitis.

## Statistical analysis

All data were summarized and displayed as mean ± SD for the continuous variables. Categorical data were expressed as frequencies and percentages.

Comparison of groups was performed using the Student t test for continuous variables normally distributed and Mann–Whitney U test for not normally distributed. The chi-square test was used to evaluate the differences for categorical variables.

An assessment of the correlation between JIA and JIA-U groups and specific variables was performed using the Pearson and Spearman rank correlation. Finally, we performed a stepwise multivariate linear regression analysis that evaluated all variables with a significant bivariate relationship (defined by *p* < 0.05) with JIA or JIA-U, as well as variables known to influence uveitis, for inclusion in the model. Statistical significance was set at p value < 0.05.

The SPSS statistical package version 25 was used to perform all statistical evaluations (SPSS Inc., Chicago, IL).

### Ethical considerations

The study complied with the Declaration of Helsinki and was approved by ethic committee.

## Results

The baseline characteristics of the present cohort are shown in Table 1 and all study samples including the past JIA-U cohort by Paroli MP in Table 2.

**Table 1:** Main baseline patients’ characteristics of the present cohort: patients with only JIA (group I) and patients with JIA-associated uveitis (JIA-U) (group II)

	**All Cohort (n = 49)**		
	**JIA (group I)** **(n = 31)**	**JIA-U (group II) ** **(n = 18)**	**p value**
**Female sex, n (%)**	20 (64.5%)	15 (83.3%)	0.140
**Oligoarthritis, n (%)**	18 (58%)	11 (61.1%)	0.834
**Age at JIA diagnosis (mean ± SD), years**	7.9 ± 4.3	3.9 ± 2.4	< 0.001
**Age at uveitis diagnosis (mean ± SD), years**	–	4.9 ± 3.0	
**Time between JIA and uveitis diagnosis (mean ± SD), years**	–	1.8 ± 2.1	
**ANA positive, n (%)**	16 (51.6%)	13 (72.2%)	0.0157
**CRP (≥ 6000 μg/L) at the time of uveitis diagnosis, n (%)**	11 (35.5%)	7 (38.8%)	0.539
**ESR (≥ 20 mm/h) at the time of uveitis diagnosis, n (%)**	13 (41.9%)	10 (55.6%)	0.253
**CRP (≥ 6000 μg/L) at 6 months follow-up from uveitis diagnosis, n (%)**	19 (61.3%)	12 (66.7%)	> 0.99
**ESR (≥ 20 mm/h) at 6 months follow-up from uveitis diagnosis, n (%)**	12 (38.7%)	10 (55.5%)	0.41

**Table 2:** Main baseline patients’ characteristics comparing the present JIA-U cohort (group II) to the JIA-U past cohort pre-dating wide-spread use of biologic therapies (group III)*

	**JIA-U present cohort (group II)** **(n = 18)**	**JIA-U past cohort (group III)** **(n = 69)**	**p value**
**Female sex**	15 (83.3%)	55 (79.7%)	0.512
**Age at JIA diagnosis (mean ± SD), years**	3.9 ± 2.4	4.0 ± 0.8	0.730
**Age at uveitis diagnosis (mean ± SD), years**	4.9 ± 3.0	3.0 ± 0.5	< 0.001
**Time interval between JIA and uveitis diagnosis (mean ± SD), years**	1.8 ± 2.1	1.2 ± 0.25	0.017
**Follow-up duration (mean ± SD), years**	5.1 ± 3.8	3.17 ± 4.3	0.086
**ANA positive**	13 (72.2%)	61 (88.4%)	0.09
**Patients on therapy at the last evaluation**	18 (100%)	69 (100%)	1.00
**Ocular topical therapy**	18 (100%)	69 (100%)	1.00
**NSAIDs**	13 (72.2%)	47 (68.1%)	0.489
**Systemic corticosteroids**	6 (33.3%)	47 (68.1%)	0.008
**csDMARDs monotherapy**	5 (27.8%)	27 (39.1%)	0.373
** csDMARDS**	15 (83.3%)	40 (58%)	0.039
**Type of medications: csDMARDs**			
MTX	14 (93.3%)	23 (57.5%)	0.035
SSZ	–	–	
AZA	1 (6.7%)	1 (2.5%)	
CsA	–	5 (12.5%)	
MTX + CsA	–	11 (27.5%)	
**bDMARDs monotherapy**	1 (5.6%)	–	–
**bDMARD + csDMARD**	10 (55.6%)	13 (18.8%)	0.003
**Type of medications: bDMARDs**			
Adalimumab	8 (72.7%)	1 (7.7%)	0.005
Infliximab	1 (9.1%)	8 (61.5%)	
Etanercept	–	3 (23.1%)	
Tocilizumab	1 (9.1%)	1 (7.7%)	
Abatacept	1 (9.1%)	–	

*Paroli MP et al. Ocul Immunol Inflamm. 2015 Feb, p. 23(1):74–81

Except where indicated otherwise, values are the number (%)

Abbreviations: JIA juvenile idiopathic arthritis; ANA antinuclear antibody; NSAIDs non-steroidal anti-inflammatory drugs; MTX methotrexate; SSZ salazopyrine; AZA azathioprine; CsA Cyclosporine; csDMARDS conventional synthetic disease-modifying anti-rheumatic drugs; bDMARDs biologic disease-modifying anti-rheumatic drugs

The present cohort included 49 children with a diagnosis of JIA according to the International League of Associations for Rheumatology criteria [12]. Patients affected from JIA associated with uveitis were 18/49 (36.7%) (On a total of 31 eyes) (group II, JIA-U) while 31/49 (63.3%) children had only a diagnosis of JIA (group I).

Overall, the present study cohort was characterized by marked female prevalence in both groups, although mostly in the group II, as well as a higher frequency of oligoarthritis and ANA positive according to ILAR criteria. In all cohort, the mean follow-up duration was 5.1 ± 3.8 years. The mean age at the JIA diagnosis was 6.3 ± 4.3 years with a JIA disease duration, at last, follow-up of 5.3 ± 3.7 years. The mean interval between JIA and uveitis diagnosis was 1.8 ± 2.1 years. A lower age at JIA diagnosis was observed in patients with associated uveitis, in particular a mean age at diagnosis of JIA less than 8 years (*p* = 0.0001). In 15/18 (83.3%) patients of the group II, the JIA diagnosis preceded the uveitis, while only in one patient (5.6%) the ocular involvement appeared one year before arthritis, and in two patients (11.1%) uveitis was detected at the same time of initial diagnosis of JIA. JIA was diagnosed mostly between 6 and 10 years of age in 38.7% of the whole cohort of 49 patients. Laboratory tests such as the mean value of (CRP) and (ESR) at uveitis’ diagnosis and at 6 months of follow-up were compared between two groups I and II, JIA and JIA-U, showing no significant differences (Table 1).

During the study period (April 2012–July 2018), a bilateral eye involvement was reported for 13/18 (72.2%) patients with a total number of 31 eyes with uveitis. The baseline characteristics of JIA-U patients (group II) and related JIA uveitis features are summarized in Table 3.

**Table 3:** JIA-U patients and uveitis characteristics comparing the JIA-U present cohort (group II) to the JIA-U past cohort pre-dating wide-spread use of biologic therapies (group III)*

	**JIA-U present cohort (group II) ** **(n = 18)**	**JIA-U past cohort (group III) ** **(n = 69)**	**p value**
**Female sex n (%)**	15 (83.3%)	55 (79.7%)	0.512
**JIA subtype and frequency of uveitis, n (%)**			
Oligoarthritis	11 (61.1%)	44 (63.8%)	0.924
Polyarthritis RF +	3 (16.7%)	9 (13%)	
Polyarthritis RF−	4 (22.2%)	16 (23.2%)	
**Uveitis involvement, n (%)**			
Unilateral	5 (27.8%)	22 (32%)	0.489
Bilateral	13 (72.2%)	47 (68%)	
Uveitis course**, n (%)			
Acute	3 (16.7%)	9 (13.0%)	0.924
Recurrent	1 (5.6%)	4 (5.8%)	
Chronic	14 (77.8%)	56 (81.2%)	
**Affected eyes, n (%)**	31 (86.1%)	116 (84.1%)	0.761
**Ocular complications, n (%)**			
Posterior synechiae	11 (35.5%)	76 (65.5%)	0.007
Cataract	2 (6.5%)	63 (54.3%)	< 0.001
Band keratopathy	3 (9.7%)	63 (54.3%)	< 0.001
Elevated IOP	2 (6.5%)	25 (21.5%)	0.047
Macular edema	4 (12.9%)	18 (15.5%)	0.505
Hypotonia	–	21 (18.1%)	–
**Ocular complications during follow-up, n (%)**			
Posterior synechiae	11 (35.5%)	16 (13.8%)	0.008
Cataract	2 (6.5%)	49 (42.2%)	< 0.001
Band keratopathy	3 (9.7%)	19 (16.4%)	0.287
Elevated IOP	2 (6.5%)	22 (19%)	0.068
Macular edema	4 (12.9%)	16 (13.8%)	0.558
Hypotonia	–	16 (13.8%)	–
**Uveitis recurrences during follow-up (mean ± SD)**	4.53 ± 4.47	31.6 ± 40.7	0.005

*Paroli MP et al. Ocul Immunol Inflamm. 2015 Feb, p. 23(1):74–81

**Uveitis Course (defined by Standardization of uveitis nomenclature 2005): acute an episode characterized by sudden onset and limited duration; recurrent repeated episodes separated by periods of inactivity without treatment ≥ 3 months in duration; chronic persistent uveitis with relapse within < 3 months after discontinuing treatment. Jabs DA et al. Am J Ophthalmol 2005, 140(3):509e16

Ocular complications were observed more frequently in oligoarticular JIA subtype and the mean age at the time of first ocular complications was 6 ± 2.7 years. Moreover, structural ocular complications were observed all at follow-up, in 8/18 (44.4%) patients and 13 eyes (41.9% of 31).

The majority of JIA-U patients (62.5%) developed only one structural ocular complication, while 12.5% and 25% of children, respectively, had two or three of them. The frequencies of ocular complications were 37.5% at 3 months, 12.5% at 6 months, and 50% at > 1 year from the uveitis diagnosis, with a mean interval time between them of 19.3 ± 18.3 months. More than two-thirds of JIA-U patients (13/18, 72.2%) were ANA positive and 6 of whom (6/13, 46.2%) developed ocular complications mainly synechiae (83.3%).

The visual prognosis of the patients examined appears favorable. In fact, at the end of the follow-up, 83.9% of eyes reached a vision of 20/20 while low vision of 20/100 was observed in only one eye of a patient with bilateral uveitis, with preservation of a vision of 20/20 in the contralateral eye.

Analyzing the data on visual acuity at the beginning and at the end of the follow-up, it emerges that of the 31 eyes affected by uveitis, 25/31 (80.6%) eyes had visual acuity between 20/20 and 20/30, 4/31 (12.9%) eyes between 20/40 and 20/70 and 2/31 (6.5%) eyes visual acuity reduced to 20/200; while at follow-up 28/31 (90.3%) eyes had vision between 20/20 and 20/30, 2/31 (6.4%) eyes between 20/40 and 20/70 and one eye (3.2%) visual equal to 20/100. During the follow-up, there was also an improvement in vision in 12 eyes with a mean of 4.5 ± 2.5 SD Snellen lines (min 2-max 9) while in 5 a worsening of 5 ± 2.6 SD Snellen lines (min 2-max 9).

15/18 (83.3%) JIA-U patients were on therapy with a csDMARD and in 10/18 (66.7%) ocular relapses were reported.

In 10/18 (55.6%) patients a combination therapy with bDMARD was needed, due to lack of uveitis remission. A total of 11 patients were on therapy with bDMARDs. Eight JIA-U patients maintained ocular remission on therapy with adalimumab. Only one patient was on monotherapy with bDMARD (adalimumab) due to methotrexate intolerance. In this patient, synechiae were described 5 years before initiation of adalimumab. Furthermore, one patient started therapy with adalimumab and then switched to infliximab to obtain ocular remission. Due to failure or loss of efficacy during anti-TNFα therapy, the use of abatacept or tocilizumab in two children was needed to achieve the uveitis’s control. Despite csDMARDs-bDMARDs combination therapy, 6/10 (60%) patients had ocular relapses.

In the present JIA-U cohort on biological medications, we observed a reduction of AC cell grade at follow-up compared to the uveitis diagnosis, specifically 8/11 (72.7%) were free of ocular inflammation at 3 months after the bDMARD introduction. BCVA also improved in 7 of 11 patients (63.6%).

Furthermore, the patients on therapy with csDMARDs plus bDMARDs showed a statistically significant higher frequency of visual acuity improvement than those treated on just csDMARDs (p = 0.042).

Therefore, the data were analyzed to compare JIA-U current cohort (group II) with the JIA-U past cohort (group III) referred to patients enrolled in the same uveitis center [14], as shown in Table 2. The past JIA-U cohort of patients presented with lower age at uveitis diagnosis and a shorter time interval between JIA and uveitis diagnosis than the current JIA-U cohort.

All patients in both JIA-U cohorts were on therapy with topical corticosteroid medications (dexamethasone eye drops, 1 to 6 drops/day, depending on severity of uveitis) and mydriatic eye drops (homatropine and/or tropicamide). Almost 70% of JIA-U past cohort used systemic corticosteroids, significantly more than the current JIA-U group (*p* = 0.008). JIA-U present cohort was on therapy more frequently with csDMARDs than the past group (*p* = 0.039), mostly treated with methotrexate (93.3%). Furthermore, a larger use of bDMARDs was described in the current JIA-U group (p = 0.005) also associated with csDMARDs (*p* = 0.003). A larger use of adalimumab (72.7%) was observed in the present JIA-U cohort compared to a greater use of infliximab (61.5%) in the past (*p* = 0.005) (Table 2).

Moreover, by comparing the JIA-U present cohort (group II) to the cohort pre-dating wide-spread use of biological therapies (group III) by Paroli MP et al. [14] (Table 3), we found a higher mean number of recurrences during follow-up in the previous cohort than the current one (31.6 ± 40.7 Vs. 4.53 ± 4.47, *p* = 0.005). As shown in Table 3 a significantly higher frequency of ocular complications was observed in the past cohort with already evidence in the first ophthalmological examination, whereas no ocular complications were present in the current study at the first observation. Only for the macular edema, no significant difference was described (*p* = 0.505), and hypotonia was not reported in the present our cohort. In the present cohort, the most common ocular complications on the affected eyes were posterior synechiae (35.5%), followed by cystoid macular edema (12.9%), band keratopathy (9.7%), and cataract (6.5%), elevated intraocular pressure (IOP) (6.5%).

During the follow-up the posterior synechiae were significantly more frequent in current JIA-U group than in the past one (p = 0.008), compared to a higher frequency of cataract in the past (< 0.001).

Overall, we observed a lower number of patients required eye surgery in the JIA-U present group compared to the previous one by Paroli MP et al.

## Discussion

Despite advances in treatment, the JIA-associated uveitis (JIA-U) is still a disabling condition, with possible long-term complications and risk of blindness [19–21].

About one-third of our JIA patients (36.7%) had associated uveitis; oligoarthritis was the most common form of JIA, affecting 59.2% of children in all our cohort and 61.1% of JIA-U, with a definite preference of females (up to 83.3% in JIA-U) although without statistical difference among JIA children with and without uveitis. A significantly higher frequency of ANA-positive patients was observed in the JIA-U group (72.2%) (p = 0.0157). Moreover, in 83.3% of our cases, the diagnosis of JIA preceded the ocular involvement, and we observed a significant correlation between uveitis onset and younger age at JIA diagnosis [13, 22, 23, 23, 24, 24, 25]. Calandra et. Al [25] reported a higher uveitis risk related to younger age at JIA onset, ANA positivity but not to the gender and Saurenmann RK et al. [26] described the patient’s age at the time of arthritis onset and ANA positivity in girls but not in boys as factors related to uveitis [27]).

In our data the mean age of JIA diagnosis in children who developed uveitis (3.9 years) and the mean interval time between JIA and uveitis diagnosis (± 20 months) were in accordance with several studies by Heiligenhaus et al. [17], Papadopoulou M. [28], Taha R. [29] and Cecchin V. [30] (specifically they reported the following data: 3.8 years/21 months, 3.4 years/17 months, 3.3 years/18 months, 4.4 years /1.6 months, respectively).

The age of first uveitis manifestation was also analyzed compared to different age groups, and the mean age at uveitis diagnosis at 5 years of age according to the study by Castagna I et al. [31]. Although, Castagna I et al. evidenced two different peaks of age for the ocular involvement, the first occurred between 4 and 6 years old and the second between 10 and 12 years old. Also, Hoeve M et al. [32] described a second peak around puberty.

Our study is a continuation of the study by Paroli MP. et al. [14] referred to patients enrolled in previous seven years in the same uveitis center in Policlinico Umberto I Sapienza.

Nevertheless, the JIA cohort in the first study was larger, but the mean follow-up was shorter than our new study (3.17 ± 4.3 years Vs. 5.1 ± 3.8 years).

A higher frequency of oligoarthritis and a female prevalence in JIA-U patients were confirmed in both studies. A longer time interval between the JIA and uveitis diagnosis was described in the present cohort (*p* = 0.017), maybe due to an early JIA diagnosis and treatment with a delay on ocular inflammation complications.

Regarding the laboratory exams, similarly to the previous study, a higher frequency of ANA positive in JIA-U patients was found: 72.2% versus 88.4% of the previous study, while the inflammation index ESR was positive at the uveitis diagnosis in 55.6% of JIA-U patients in this study compared to 76.5% of the previous one. In the study by Paroli MP et al., 30% of affected eyes had at least one ocular complication at first visit, while in this study no patients presented ocular complications at first ophthalmological evaluation and the majority (50%) developed them at > 1 year from the uveitis diagnosis, with a mean interval time between them of 19.3 ± 18.3 months.

In this study the frequency of ocular complications observed was lower compared to the previous, respectively: posterior synechiae (35.5% and 65.5%), cataract (6.5% and 54.3%), band keratopathy (9.7% and 54.3%), elevated IOP (6.5% and 21.5%), and the macular edema (12.9% and 15.5%).

The hypotonia, considered as one of the most frequent cause of low vision and blindness in the past, was not described in our study.

The data about the late and lower development of ocular complications such as posterior synechiae, cataract, and band keratopathy could be due to an early diagnosis and the ophthalmological screening for JIA in the last decade.

The limitations of the present study could be the relatively small population and the retrospective design. Nevertheless, the main strength of the study is the comparison between a present JIA-U cohort with a previous JIA-U group of patients enrolled before the wide-spread use of biological therapy in the same uveitis center.

In the past cohort by Paroli MP et al. [14] many patients came from rheumatological centers from different parts of Italy or directly from ophthalmologists with an inevitable delay in JIA diagnosis, while the close collaboration with the pediatric rheumatologic center in the same University has contributed to the care improvement and decrease of ocular complications. Moreover, the reduction and prevalence of the uveitis severity in JIA patients could be related to greater knowledge about JIA uveitis, an early treatment and the introduction of new therapies including biological agents. In fact, the treatment with systemic corticosteroid therapy was significantly decreased in our current cohort compared to the past one (*p* = 0.008) for the use of csDMARDs and bDMARDs, being another possible cause of reductions of cataracts in the most recent cohort.

The importance of early administration of biologic therapy and its role to improve also extraocular manifestations, it has been highlighted also in other forms of pediatric uveitis such as in Behçet’s disease, typically managed by topical corticosteroids and mydriatic agents [33, 34].

Furthermore, in our present JIA-U cohort on biological therapy, a higher visual acuity improvement was observed, according to data reported to other authors also in other pediatric uveitis of diverse etiologies [35, 36]

The likely different response rates to biological therapy between non-infectious pediatric anterior uveitis of different aetiologies [36], as well as treated with anti- TNF-α agents, infliximab or adalimumab, have been described in the literature [37, 38]. Additional comparison studies are needed to clarify all possible differences.

## Conclusion

Although new drugs improved significantly the course of JIA and visual prognosis of associated uveitis, the disease remains a challenge for pediatric rheumatologists and ophthalmologists, and patients are still developing sight-threatening ocular complications. A routine ophthalmological follow-up is therefore required at regular intervals, in order to identify early ocular involvement.

Further studies are needed to better characterize a target therapeutic approach. The appropriate treatment is necessary to reduce the long-term disability and improve the quality of life in JIA-U.

Optimal care of children with JIA and uveitis requires careful and close collaboration between ophthalmologists and rheumatologists to choose the most appropriate systemic therapy. New discoveries on the pathogenesis of JIA-U and the identification of predictive biomarkers could help find a targeted therapy tailored to individual patients and based on their predicted response or risk of disease.
